# *In Vitro* Bioconversion of Pyruvate to *n*-Butanol with Minimized Cofactor Utilization

**DOI:** 10.3389/fbioe.2016.00074

**Published:** 2016-10-17

**Authors:** Steven Reiße, Martina Haack, Daniel Garbe, Bettina Sommer, Fabian Steffler, Jörg Carsten, Frank Bohnen, Volker Sieber, Thomas Brück

**Affiliations:** ^1^Department of Chemistry, Technical University of Munich, Garching, Germany; ^2^B&B Sustainable Innovations GmbH, Köln, Germany; ^3^Straubing Center of Science, Technical University of Munich, Straubing, Germany

**Keywords:** butanol, cell-free, enzyme cascade, biocatalysis, chemoenzymatic, synthetic biotechnology

## Abstract

Due to enhanced energy content and reduced hygroscopicity compared with ethanol, *n*-butanol is flagged as the next generation biofuel and platform chemical. In addition to conventional cellular systems, *n*-butanol bioproduction by enzyme cascades is gaining momentum due to simplified process control. In contrast to other bio-based alcohols like ethanol and isobutanol, cell-free *n*-butanol biosynthesis from the central metabolic intermediate pyruvate involves cofactors [NAD(P)H, CoA] and acetyl-CoA-dependent intermediates, which complicates redox and energy balancing of the reaction system. We have devised a biochemical process for cell-free *n*-butanol production that only involves three enzyme activities, thereby eliminating the need for acetyl-CoA. Instead, the process utilizes only NADH as the sole redox mediator. Central to this new process is the amino acid catalyzed enamine–aldol condensation, which transforms acetaldehyde directly into crotonaldehyde. Subsequently, crotonaldehyde is reduced to *n*-butanol applying a 2-enoate reductase and an alcohol dehydrogenase, respectively. In essence, we achieved conversion of the platform intermediate pyruvate to *n*-butanol utilizing a biocatalytic cascade comprising only three enzyme activities and NADH as reducing equivalent. With reference to previously reported cell-free *n*-butanol reaction cascades, we have eliminated five enzyme activities and the requirement of CoA as cofactor. Our proof-of-concept demonstrates that *n*-butanol was synthesized at neutral pH and 50°C. This integrated reaction concept allowed GC detection of all reaction intermediates and *n*-butanol production of 148 mg L^−1^ (2 mM), which compares well with other cell-free *n*-butanol production processes.

## Introduction

*n*-Butanol is a primary 4-carbon alcohol, which is flagged as the next generation biofuel and platform chemical due to its enhanced energy content and reduced hygroscopicity compared with ethanol (Li et al., 2010). *n*-Butanol is of interest as a platform chemical in the chemical, textile, polymer, and biofuel industry (Dürre, 2007).

Conventionally, biotechnological *n*-butanol production is based on the anaerobic ABE fermentation process, which utilizes solventogenic *Clostridia* strains, such as *Clostridium acetobutylicum* (Lin and Blaschek, 1983; Vollherbst-Schneck et al., 1984; Dusseaux et al., 2013). However, this process involves formation of several undesired by-products (i.e., butyrate) (Lee et al., 2008).

In contrast to cell-based production systems, the recent application of artificial, solvent tolerant enzyme cascades provides opportunities for targeted *in vitro* biobutanol production that allow operation at higher process temperatures. Exemplary, we previously established a modular, *in vitro* enzyme cascade that allows the flexible conversion of glucose to either ethanol or isobutanol at 50°C, respectively (Guterl et al., 2012). This enzyme system is based on an artificial glycolytic reaction cascade that utilizes pyruvate as the central intermediate.

In contrast to the *in vitro* ethanol and isobutanol production system, the design of an *n*-butanol-specific enzyme cascade is more complex and involves several bottlenecks, multiple coenzyme A (CoA)-dependent intermediates and unstable cofactors. The natural clostridial *n*-butanol biosynthesis from glucose comprises 16 enzyme activities, 5 CoA-dependent intermediates, 3 cofactors, and several ATP-dependent conversion steps (Inui et al., 2008; Bar-Even et al., 2012; Krutsakorn et al., 2013). At present, an *in vitro* enzyme cascade for *n*-butanol production from glucose has been described, which in analogy to the cell-based system involves 16 different enzyme activities for glycolysis and an artificial solventogenesis utilizing thermostable enzyme variants (Krutsakorn et al., 2013). Notably, the reaction cascade primarily focused on converting pyruvate to *n*-butanol utilizing seven enzyme activities, CoA and NADH as cofactors. In this approach, unbalanced concentrations of CoA-dependent intermediates and generated NAD^+^ inhibit crucial enzyme activities, such as a crotonase similar 3-hydroxypropionyl-CoA dehydratase, thiolase, and β-hydroxybutyryl-CoA dehydrogenase, thereby limiting product yield (Engel et al., 1996; Sommer et al., 2013a; Reisse et al., 2014). In order to sustain *in vitro n*-butanol biosynthesis in the presence of CoA and NAD^+^, therefore, required a time-dependent feeding of metabolic intermediates to overcome enzyme-specific feedback inhibition and temperature-dependent cofactor instability (Krutsakorn et al., 2013). In general, managing the cofactor balance and adjusting a linear production rate is very difficult considering that complete inhibition of a single enzyme activity would arrest the entire process. Furthermore, ATP is unstable under extended process times and at elevated temperatures. In addition, controlling the stoichiometry of various CoA-dependent intermediates requires constant feeding during the reaction, which increases process complexity and cost.

In this study, we have focused on establishing an *in vitro n*-butanol production process from pyruvate at elevated temperatures. This cell-free process is minimized with respect to employed enzyme activities. In comparison to previous studies, we achieved to entirely eliminate all ATP and CoA-dependent reactions using NADH as the sole redox shuttle. Our study has focused on conversion of pyruvate to *n*-butanol, as there are now several efficient cell-based (Xu et al., 2008) and *in vitro* routes (Gao et al., 2009; Guterl et al., 2012) for the production of this platform metabolite available. We have chosen a reaction temperature of 50°C to be compatible with our previous study describing the cell-free conversion of glucose to pyruvate (Guterl et al., 2012).

**Figure 1:**
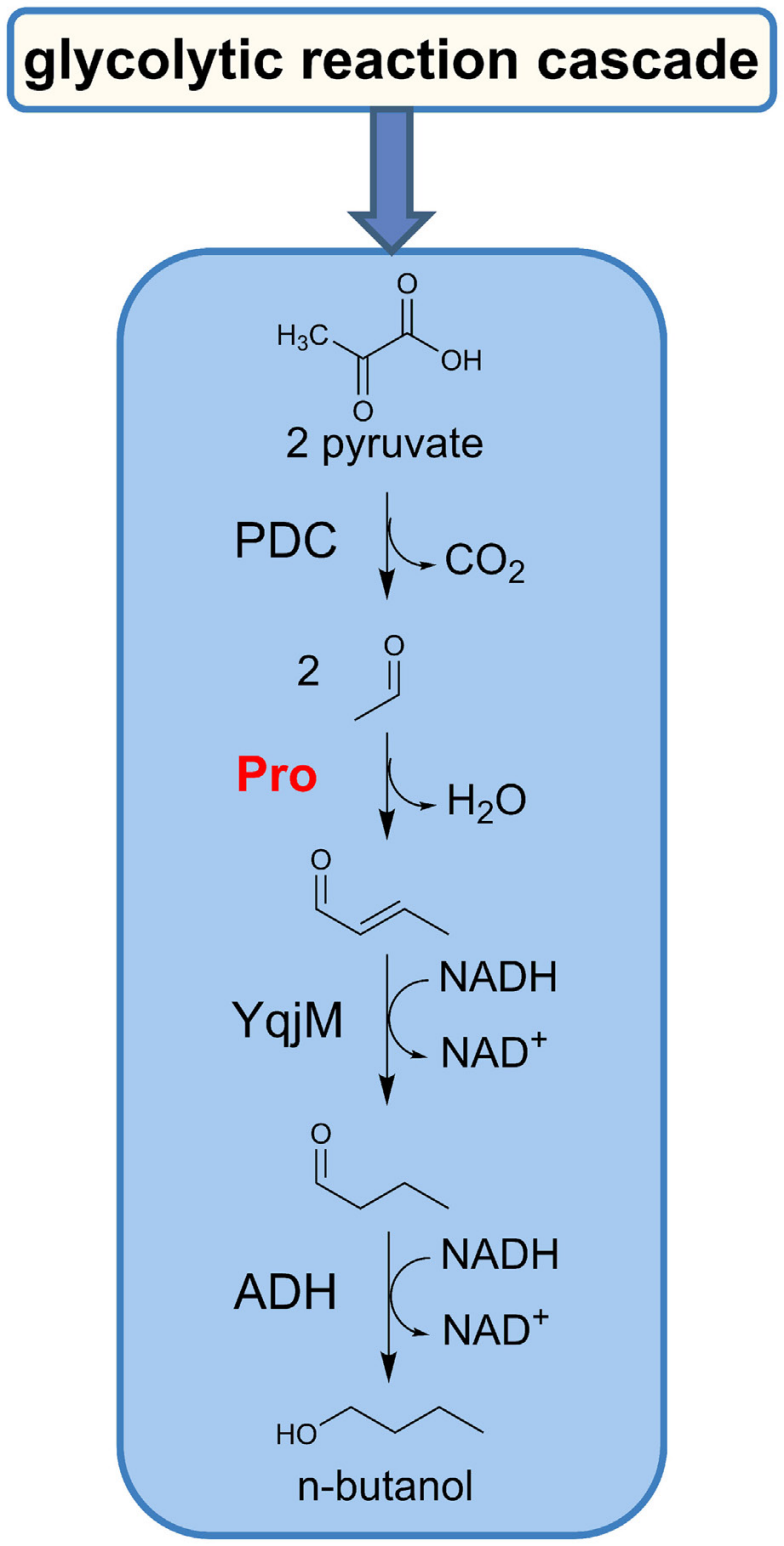
**Schematic illustration of the *n*-butanol reaction cascade *via* the enamine–aldol condensation**. PDC, pyruvate decarboxylase; Pro, proline; YqjM, 2-enoate reductase; ADH, alcohol dehydrogenase.

The realization of our *in vitro* biocatalytic reaction cascade predominantly relies on the substitution of CoA-dependent reaction steps by an organocatalyst, such as an amino acid or diamine, which facilitates the direct conversion of acetaldehyde to crotonaldehyde by an enamine–aldol condensation (List, 2002). The implementation of this enamine condensation now enables the bypass of the native *n*-butanol pathway enzymes, such as CoA-acetylating aldehyde dehydrogenase, thiolase, hydroxybutyryl-CoA dehydrogenase, crotonase, and butyryl-CoA dehydrogenase. This approach provides direct access to an alternative *n*-butanol cascade, which we postulated previously (Sommer et al., 2013b). The resulting *in vitro* biocatalytic reaction cascade is the first report of an artificially designed thermotolerant and highly condensed *n*-butanol biosynthesis route (Figure 1).

**Figure 2:**
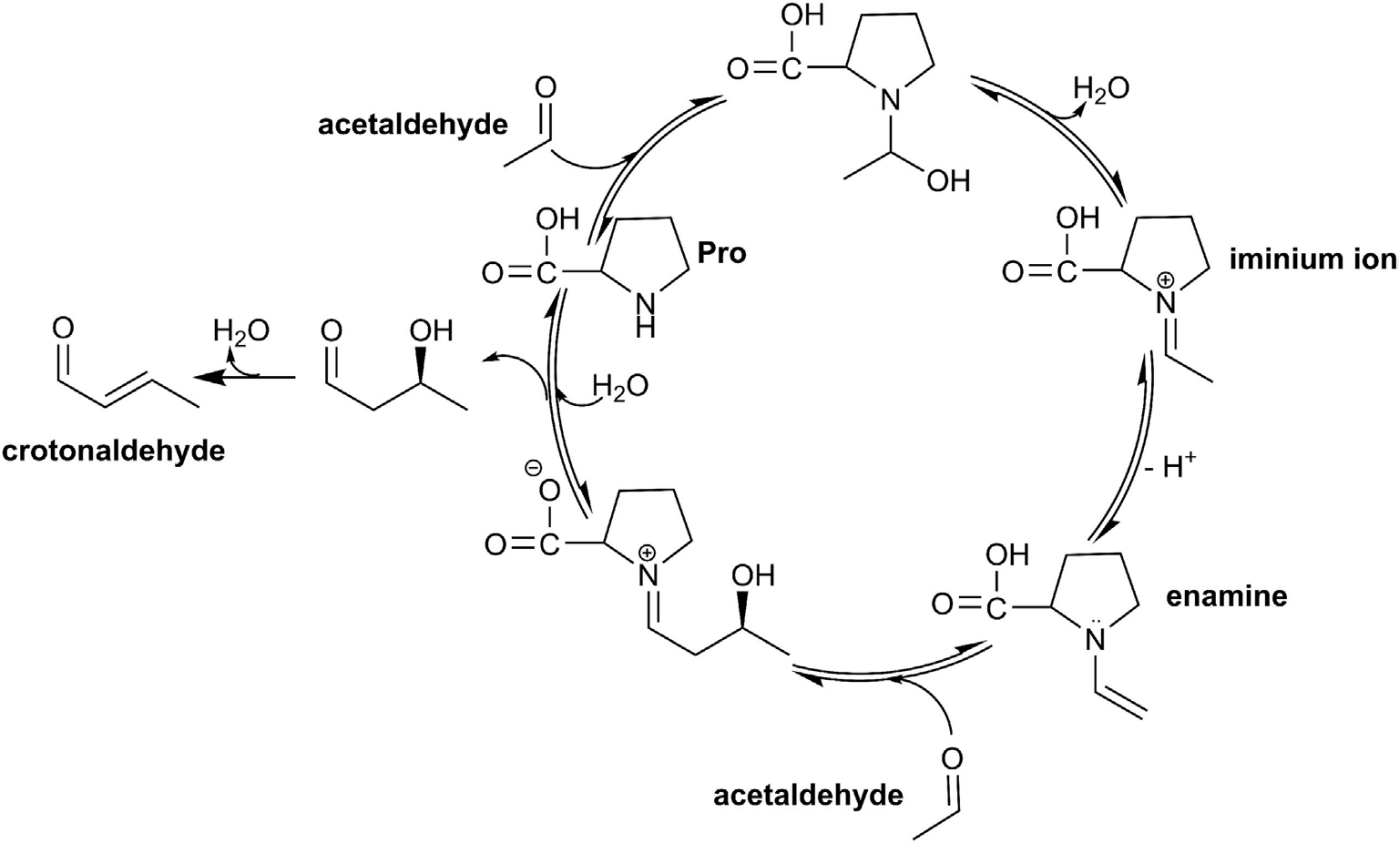
**Catalytic cycle of the enamine–aldol condensation of acetaldehyde, applying the catalyst proline**.

The primary conversion involves the pyruvate decarboxylase (PDC) to acetaldehyde, which in turn serves as a substrate for the biochemically catalyzed enamine–aldol condensation, utilizing amino acids (i.e., proline) or natural diamines as a catalyst (Figure 2).

In this respect, proline is unique due to its pyrrolidine moiety, which translates to a pronounced nucleophilic reactivity. While the pyrrolidine moiety readily forms iminium ions and enamines (List, 2002), amino acids, such as arginine or tryptophan, featuring a secondary amine moiety can serve as suitable catalysts. Moreover, natural polyamines, such as spermidine or putrescine, show the desired catalytic capacity albeit at lower reactivity compared with the former (Theruvathu et al., 2005).

In a subsequent two-step reaction, the resulting crotonaldehyde is converted to butyraldehyde and finally to *n*-butanol by action of a 2-enoate reductase (YqjM) and an alcohol dehydrogenase (ADH) (Sommer et al., 2013b).

Consequently, our designed biocatalytic *n*-butanol production cascade is consolidated to use only three enzyme activities, an amino acid with secondary amine functionality and NADH as reducing equivalent. With reference to previously reported *in vitro n*-butanol reaction cascades, we have eliminated five enzyme activities, the requirement of ATP and CoA as a cofactor as well as five CoA-dependent intermediates.

## Materials and Methods

### Chemicals

All chemicals were purchased in analytical grade from Sigma-Aldrich (Munich, Germany) and Carl Roth (Karlsruhe, Germany).

### Enzyme Selection

Pyruvate decarboxylase (*Zymomonas mobilis*) and YqjM (*Bacillus subtilis*) were selected due to their established catalytic performance at the desired reaction conditions (50°C, neutral pH). Moreover, both enzyme systems have previously been used in our cell-free solvent production cascades (Guterl et al., 2012; Sommer et al., 2013b). As our experimental approach focused on the application of non-commercial enzymes, we have integrated an alternative ADH from *Geobacillus stearothermophilus*. Data characterizing this enzyme are presented in this study.

### Cloning

The cloning procedures for plasmids containing PDC and YqjM followed literature protocols (Guterl et al., 2012; Sommer et al., 2013b). A codon-optimized variant of the ADH gene (*ADH-HT*, NCBI accession number: CAA81612.1) was synthesized by Life Technologies (Regensburg, Germany). The artificial gene was cloned into the *Escherichia coli*-compatible vector pET-22b (Merck, Darmstadt, Germany) with C-terminal His-Tag *via Nde*I and *Xho*I restriction sites.

### Enzyme Preparation

Enzyme expression was performed using *E. coli* BL21 (DE3) or HMS173 (DE3) (Novagen, Nottingham, UK) as host strains. Cultivations were carried out in shake flask cultures or in a 10-L Biostat C plus bioreactor (Sartorius Stedim, Göttingen, Germany). Recombinant cells were initially cultivated at 37°C in TB medium supplemented with kanamycin (30–50 μg mL^−1^), unless otherwise stated. The cells were induced with 1 mM isopropyl-β-d-thiogalactopyranoside (IPTG) at OD_600_ 0.5–0.8. Cultures were subsequently harvested and frozen at −20°C until further use. PDC was expressed in Zyp-5052 (Studier, 2005), YqjM in TB medium. After induction with IPTG, growing temperatures were lowered at 30 or 25°C. ADH was expressed in a fed-batch cultivation method at 37°C using LB as medium supplemented with 0.25 mM ZnSO_4_ and 100 μg mL^−1^ ampicillin. The cells were induced with 0.3 mM IPTG at OD_600_ = 5 and further cultivated for 4 h before harvest and storage at −20°C.

Cell lysates were prepared with an Emulsiflex-B15 (Avestin, Mannheim, Germany). Cell debris and protein aggregates were separated from the soluble fraction by centrifugation (21,000 × g, 4°C, 20 min). The enzymes were purified (50 mM HEPES buffer, pH 7) *via* immobilized metal affinity chromatography (IMAC) using a NGC FPLC-system (Bio-Rad, Munich, Germany) equipped with a HisTrap FF column (GE Healthcare, Freiburg, Germany). Purified enzyme solutions were desalted using a HiPrep 26/10 Desalting-column (Bio-Rad) in 50 mM HEPES pH 7 plus 10% glycerol for subsequent storage at −80°C.

### Enzyme Assays

#### Baseline Spectrophotometric ADH Characterization

The baseline characterization of ADH was conducted photometrically in 96-well microtiter plate format using a Varioskan photometer (Thermo Scientific, Braunschweig, Germany). Reactions using NADH as cofactor could be monitored at 340 nm (molar extinction coefficient NADH = 6.22 L mmol^−1^ cm^−1^). Activities were measured by following the NADH-dependent reduction of aldehydes to the corresponding alcohols in 100 mM HEPES buffer (pH 7) and 2.5 mM MgSO_4_ at 50°C, unless otherwise stated.

Optimum temperature and pH were determined by monitoring reduction rates of 1 mM acetaldehyde with 0.3 mM NADH at different temperatures (30–70°C) and in different buffers (sodium phosphate pH 5.5–6.5, HEPES pH 6.5–7, and TRIS pH 7–8), respectively. Thermal stability was tested by measuring ADH activity after different incubation times (0–24 h) at 50, 60, and 70°C. ADH kinetic parameters were determined with 0.3 mM NADH and varying concentrations of acetaldehyde (0.01–0.14 mM) or butyraldehyde (0.8–3 mM).

#### Enzyme Assays for *n*-Butanol Cascade Design

For the reaction cascade design, all enzyme activities (PDC, YqjM, and ADH) were determined under comparable reaction conditions and analyzed using a Thermo Scientific Trace GC Ultra gas chromatograph with flame ionization detector (FID). Reaction mixtures were incubated in a 2-mL GC-Vial at 50°C in a water bath for accurate temperature control, whereby the pH was adjusted to the corresponding temperature. The reaction was stopped at several time points with 7% trichloroacetic acid (TCA).

##### PDC Assay

The PDC was measured in an assay mixture containing 50 mM HEPES buffer (pH 7), 20 mM sodium pyruvate, 2.5 mM MgSO_4_, and 0.1 mM thiamine pyrophosphate (TPP). The reaction products were analyzed by GC measurements.

##### YqjM Assay

2-Enoate reductase activity was measured in a coupled assay using a horse liver ADH (Evocatal GmbH, Monheim, Germany). The reaction was carried out at 50°C (*t* = 30 min) in 50 mM HEPES buffer (pH 7) containing 2.5 mM MgSO_4_, 0.05 mM FMN, and 20 mM NADH as cofactors and 20 mM crotonaldehyde. The reaction products were analyzed by GC measurements.

##### ADH Assay

Alcohol dehydrogenase activity was determined in 50 mM HEPES buffer (pH 7) with 2.5 mM MgSO_4_ and 20 mM butyraldehyde or acetaldehyde.

One unit of enzyme activity corresponds to the amount of enzyme, which reduces 1 μmol substrate per minute.

### Enamine–Aldol Condensation

Potential catalysts for the enamine–aldol condensation were screened with a dose of 20 mM in 2 mL GC vials containing 20 mM acetaldehyde and 50 mM HEPES buffer (pH 7). The vials were incubated in a water bath at 50°C for up to 3 h and subsequently analyzed *via* GC. The reactions were stopped with 7% TCA.

In particular, the proline reaction was thoroughly examined with respect to its temperature (40–90°C), pH (5.5–10), and concentration (1–500 mM) dependency. The pH dependency was measured either in a 50 mM MES (pH 5.5–6.5), HEPES (pH 7–8), TAPS (pH 9), or CAPS (pH 10) buffer system, respectively.

### Baseline Characterization and Optimization of the Reaction System

Required enzyme quantities for the cell-free *n*-butanol production were calculated based on the previous reference measurements for individual unit activities: PDC = 72.8 ± 0.9 U mL^−1^ (toward pyruvate), YqjM = 1.1 ± 0.1 U mL^−1^ (toward crotonaldehyde), ADH = 4592 ± 65.7 U mL^−1^ (toward acetaldehyde), and 183.1 ± 3.7 U mL^−1^ (toward butyraldehyde). The proline dose was calculated based on previous control experiments carried out with different proline concentrations.

#### Optimizing the Reaction System

To determine the optimal biocatalyst composition, various reaction designs were examined. All reaction mixtures comprise 20 mM pyruvate, 0.05 mM FMN, 20 mM NADH, 2.5 mM MgSO_4_, and 0.1 mM TPP in 50 mM HEPES buffer (pH 7). To determine the optimal enzyme activity ratios different reaction designs were examined: *Reaction 1*: 0.25 U mL^−1^ PDC, YqjM, ADH, and 0.5 U mL^−1^ proline; *Reaction 2*: 0.5 U mL^−1^ PDC, proline, and 0.25 U mL^−1^ YqjM, ADH; *Reaction 3*: 0.5 U mL^−1^ PDC, YqjM, ADH, and proline; *Reaction 4*: 0.5 U mL^−1^ PDC, YqjM, ADH, and 0.25 U mL^−1^ proline. Reactions were carried out at 50°C without stirring over 10 h.

Starting with pyruvate, the whole production cascade was eventually screened in triplicate in 2 mL GC vials. All required biocatalysts and their ligands are stated in Table 1. The reaction mixture contained 20 mM pyruvate, 319 mM proline, 0.05 mM FMN, 20 mM NADH, 2.5 mM MgSO_4_, and 0.1 mM TPP in 50 mM HEPES buffer (pH 7). The final acetaldehyde conversion rate was set to 0.25 U mL^−1^. To initiate the reaction, an enzyme mixture containing 0.5 U mL^−1^ PDC, 0.5 U mL^−1^ YqjM, and 0.5 U mL^−1^ ADH (toward butyraldehyde) was added to each vial. Control reactions were performed without enzyme addition. The vials were placed in a water bath and incubated at 50°C without stirring. Samples were collected at constant 2 h time intervals over 10 h. Reactions were stopped for GC analysis by addition of 7% TCA. Samples for high-performance liquid chromatography (HPLC) analysis were pretreated by filtration (10 kDa MWCO Zentrifugal Filter, VWR, Darmstadt, Germany). Collected samples were subsequently analyzed *via* GC and HPLC to determine the product and intermediates.

**Table 1:** **Required biocatalysts to generate *n*-butanol from pyruvate**.

#	Biocatalyst	EC #	Substrate	Product
1	Pyruvate decarboxylase (PDC)	4.1.1.1	Pyruvate	Acetaldehyde
2	Proline	–	Acetaldehyde	Crotonaldehyde
3	2-enoate reductase (YqjM)	1.3.1.31	Crotonaldehyde	Butyraldehyde
4	Alcohol dehydrogenase (ADH)	1.1.1.1	Butyraldehyde	*n*-Butanol

### Analytical Methods

Aldehydes and alcohols were separated and quantified by gas chromatography using Trace GC Ultra (Thermo Scientific, Braunschweig, Germany), equipped with Headspace Tri Plus autosampler, an agitator and FID. All compounds were separated *via* a Stabilwax column (length 30 m, 0.25 mm internal diameter, 0.25 μm film thickness; Restek, Bad Homburg, Germany), with helium (1.2 mL min^−1^) as carrier gas. The oven temperature was programed to be held at 50°C for 2 min, raised with a ramp of 10 to 200°C min^−1^ and held for 1 min. Injector and detector were kept at 200°C. Samples were incubated prior to injection at 40°C for 15 min. For the analysis, 700 μL of the headspace were injected (headspace syringe 100°C) in the split mode with a flow of 10 mL min^−1^.

Pyruvate was separated and quantified *via* HPLC, using an Ultimate-3000 HPLC-system (Thermo Scientific, Braunschweig, Germany), equipped with an autosampler, a thermostatic column compartment, and a diode-array detector. The separation was achieved on a Metrosep Supp A16 column (25 mm, particle size 4.6 μm; Metrohm, Filderstadt, Germany) at 65°C by isocratic elution with 12 mM ammonium bicarbonate (pH 10), followed by a washing step with 30 mM sodium carbonate (pH 10.4). Mobile phase flow was adjusted to 0.2 mL min^−1^.

## Results

### Evaluation of Potential Catalysts for the Enamine–Aldol Condensation

Potential catalysts for the desired enamine condensation were selected and tested with acetaldehyde in 50 mM HEPES buffer (pH 7) at 50°C for maximal 3 h. The selected panel of natural organocatalysts comprises the amino acids arginine and proline as well as the polyamine spermidine. The amino acid alanine served as a negative control. While proline (List, 2002) was reported to harbor the desired nucleophilic reactivity, arginine was selected due to the secondary amine function that comprises its side chain. As spermidine also possesses secondary amines, it also represents potential catalytic candidate (Theruvathu et al., 2005).

**Figure 3:**
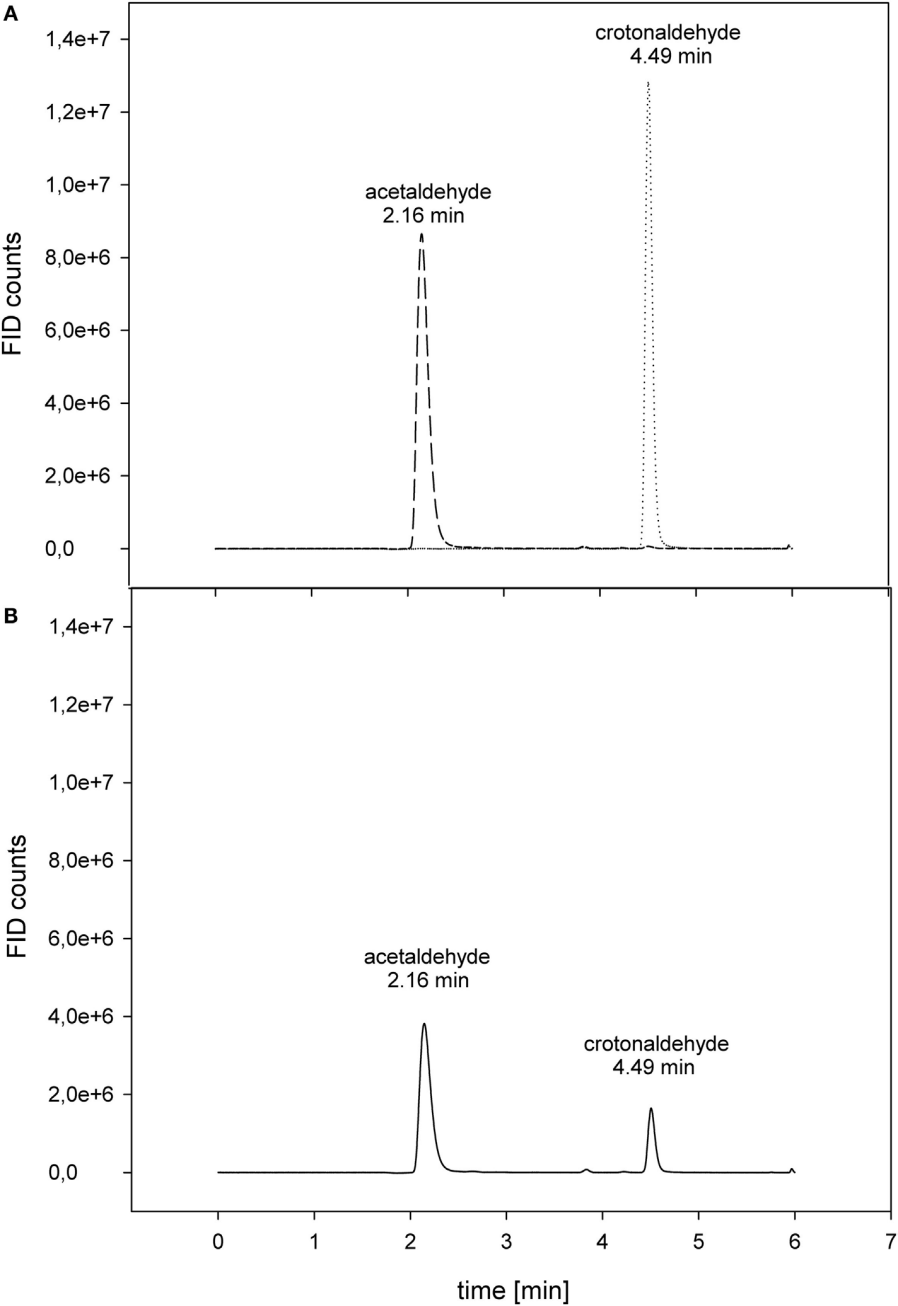
**Conversion of acetaldehyde to crotonaldehyde *via* proline**. **(A)** Separate control measurements: - - 20 mM acetaldehyde; ······ 20 mM crotonaldehyde. **(B)** Crotonaldehyde biosynthesis. The reaction (–––) was carried out at 50°C in 50 mM HEPES buffer (pH 7) with 20 mM acetaldehyde as substrate.

Proline displayed the best conversion rate of acetaldehyde to crotonaldehyde with 5.7 ± 0.2 × 10^−3^ μmol min^−1^ mL^−1^ (Figure 3). With respect to the proline reaction rate, arginine and the polyamine spermidine accomplished 46% of the reference value. As expected, the negative control alanine showed only 4% of the proline activity (Table 2).

**Table 2:** **Conversion rates for the transformation of acetaldehyde to crotonaldehyde**.

Organocatalyst	*v* (μmol min^−1^ mL^−1^)
Proline	5.7 ± 0.2 × 10^−3^
Arginine	2.6 ± 0.1 × 10^−3^
Spermidine	2.6 ± 0.0 × 10^−3^
Alanine	0.2 ± 0.0 × 10^−3^

*All organocatalysts were tested at a concentration of 20 mM at 50°C in 50 mM HEPES buffer (pH 7), containing 20 mM acetaldehyde*.

### Optimizing Reaction Conditions

To establish the new *n*-butanol cascade proline was selected and further examined regarding temperature, pH, and concentration. Thereby, our standard reaction conditions (20 mM proline, 50 mM HEPES, pH 7 at 50°C) were adapted to the corresponding experiments.

**Figure 4:**
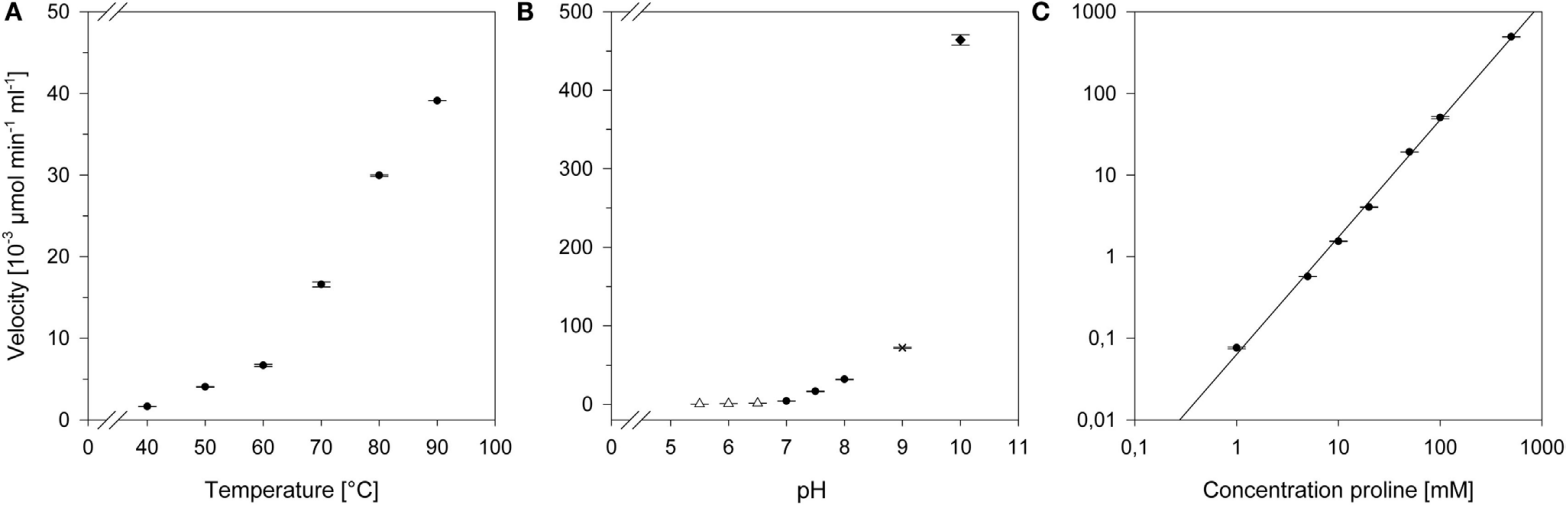
**Effects of different conditions on the proline activity**. **(A)** Temperature profile: the reactions were carried out at described conditions in a range from 40 to 90°C. **(B)** pH profile: the reactions were carried out at described conditions with following buffers: Δ, 50 mM MES buffer, pH 5.5–6.5; ●, 50 mM HEPES, pH 7–8; ×, 50 mM Taps, pH 9; ◆, 50 mM CAPS, pH 10. **(C)** Concentration-dependent activity: aldol condensation was determined in presence of various proline concentrations ranging from 1 to 500 mM. The scale is logarithmic.

The operational temperature dependency was determined by incubating the proline reaction mix between 40 and 90°C. The formation of crotonaldehyde was subsequently monitored *via* GC-FID. As illustrated in Figure 4A, the proline activity was simultaneously rising with the temperature and could be increased tenfold from 50 to 90°C.

Determination of pH effects on the proline reactivity were studied at 50°C. The proline activity over a broad pH range was evaluated with each 50 mM MES (pH 5.5–6.5), HEPES (pH 7–8), TAPS (pH 9), and CAPS (pH 10) buffer, respectively. We could measure a significant increase in activity with increasing pH (Figure 4B). Notably, the proline activity at pH 10 was over 100-fold higher than at neutral pH.

As the organocatalyst concentration is a key process parameter, we have determined the aldol condensation in the presence of increasing proline concentrations (Figure 4C). The highest detectable proline activity was 494.6 ± 4.6 × 10^−3^ μmol min^−1^ mL^−1^ at 500 mM. With the provided data, proline could be set to specific conversion rates to achieve an optimal *n*-butanol production.

Although the proline-catalyzed aldol condensation favors higher pH and temperature values, the overall reaction conditions were set to 50°C and neutral pH with respect to the enzymes characteristics of ADH, PDC, and YqjM. While ADH accepted higher temperatures over 60°C (Table 3), PDC and YqjM preferred temperatures in the range of 50°C or less. All three enzymes additionally operated optimal at neutral or slightly acidic conditions (Gocke et al., 2007; Guterl et al., 2012; Sommer et al., 2013b).

**Table 3:** ***Geobacillus stearothermophilus* ADH characteristics**.

Parameter	Value
Optimum temperature	60°C
Thermal stability (50°C)	*t*/2 ≥ 24 h
Thermal stability (60°C)	*t*/2 = 12 h
Thermal stability (70°C)	*t*/2 = 2 h
Optimum pH	6
pH range	5.5–8
*K*_m_^a^ acetaldehyde/butyraldehyde	0.065 ± 0.005 mM/1.43 ± 0.16 mM
*k*_cat_ acetaldehyde/butyraldehyde	141.44 ± 14.52 s^−1^/49.16 ± 3.8 s^−1^
*k*_cat_/*K*_m_ acetaldehyde/butyraldehyde	2184 ± 393 mM^−1^ s^−1^/34 ± 7 mM^−1^ s^−1^

*^a^The kinetic constants were measured with appropriate concentrations of NADH and various concentrations of the corresponding substrate (acetaldehyde: 0.01–0.14 mM/butyraldehyde 0.8–3 mM) in 100 mM HEPES pH 7 at 50°C*.

Considering this pathway composition and the upstream glycolytic reaction module (Guterl et al., 2012), the chosen reaction parameters represent a feasible compromise that maintains activity for all required catalytic components albeit not operating at their individual optimum.

### *In Vitro n*-Butanol Synthesis

The consolidated conversion of pyruvate to *n*-butanol was carried out at 50°C for 10 h and neutral pH conditions. Proline was applied as an organocatalyst for the desired enamine–aldol condensation due to its superior reaction rate compared with other evaluated amines. All reaction intermediates and products were quantified with authentic standards using established GC and HPLC protocols.

Initially, various reaction designs were examined in single tests to identify the optimal organo-/biocatalyst composition. Therefore, different enzyme and proline activities were evaluated and compared, as illustrated in Figure S1 in Supplementary Material. The GC data indicated that our designed *in vitro* biocatalytic reaction cascade allows the direct conversion of pyruvate to *n*-butanol. The best result of 2 mM *n*-butanol was obtained by the combination of 0.5 U mL^−1^ PDC, YqjM, ADH, and 0.25 U mL^−1^ proline.

**Figure 5:**
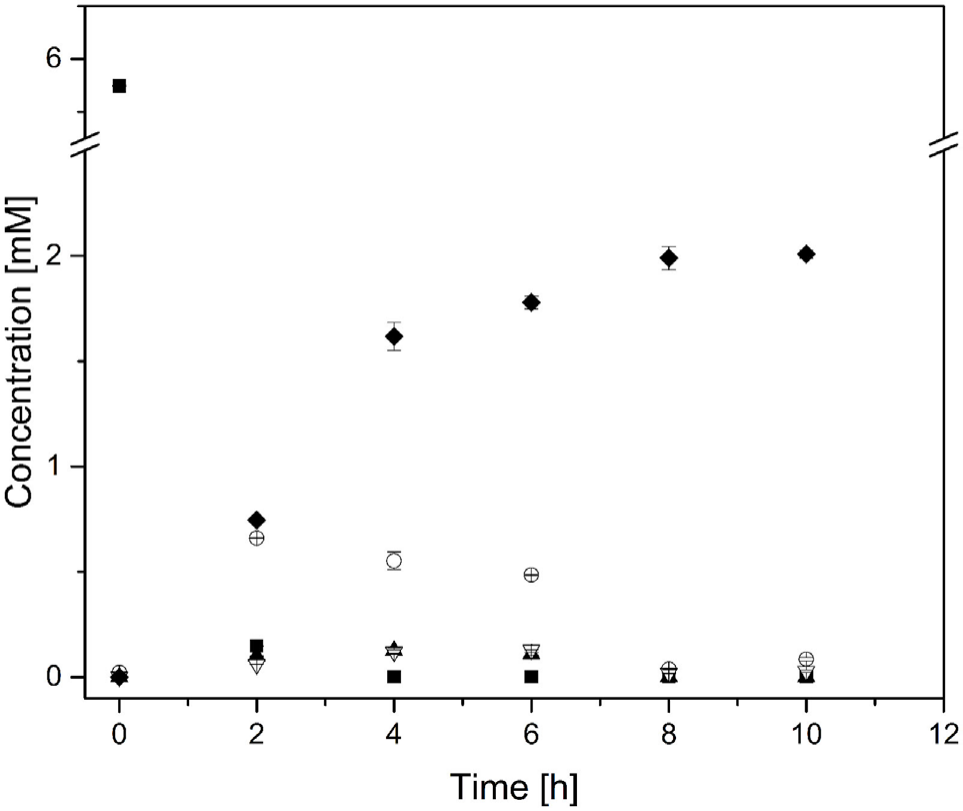
***n*-Butanol biosynthesis *via* the proline-catalyzed enamine–aldol condensation**. *n*-Butanol production time profile. The reaction was carried out in 2 mL GC vials, without stirring at 50°C and neutral pH. ■, Pyruvate; ○, Acetaldehyde; ▲, Crotonaldehyde; ▽, Butyraldehyde; ◆, *n*-Butanol. Note that the concentration of pyruvate and acetaldehyde was halved due to a better comparison with *n*-butanol concentration (2 mol pyruvate is converted to 1 mol *n*-butanol).

Subsequently, the reaction cascade was then tested in triplicate, whereby each independent experiment was triggered by addition of the enzyme mix leading to the formation of 2.0 ± 0.0 mM *n*-butanol (Figure 5). The *n*-butanol production rate remained constant during the first 4 h and achieved a production rate of nearly 0.01 μmol min^−1^ mL^−1^, which is comparable to a different cell-free approach reported by the Ohtake group (Krutsakorn et al., 2013). After 4 h, the *n*-butanol production rate decreased and finally sized at 8 h. En route to *n*-butanol formation we could detect acetaldehyde, crotonaldehyde, and butyraldehyde as intermediates, which were almost completely consumed after 8 h.

In addition to *n*-butanol, we could also detect significant ethanol formation (*c* = 4.9 ± 0.0 mM) as a reaction byproduct. As shown in Table 3, ADH showed a strong substrate preference for acetaldehyde over butyraldehyde, which lead to an unfavorable substrate flux. Hence, the ADH reaction limited the acetaldehyde and the subsequent intermediate pools.

## Discussion

The focus of this study was the design of an *in vitro* thermostable, biocatalytic reaction cascade that provides for the conversion of pyruvate to *n*-butanol with minimal enzyme activities and in the absence of any CoA-dependent intermediates. What is more, the reaction cascade should be modularly compatible with our previously reported *in vitro* production toolbox for the production of industrial solvents (Guterl et al., 2012).

This toolbox utilizes an artificial glycolytic reaction cascade. While the *in vitro* production of ethanol and isobutanol have been successfully reported previously (Guterl et al., 2012), designing a cell-free enzyme cascade for *n*-butanol was challenging particularly with respect to harmonizing enzyme activities and cofactor concentrations.

To circumvent these limitations and reduce required enzyme activities, we designed a new biocatalytic *n*-butanol pathway. Our consolidated *n*-butanol production pathway operates with only three enzyme activities, NADH as the sole redox mediator and an amino acid catalyzed enamine–aldol condensation as the essential biocatalytic component that enables a C–C bond formation leading to the required C4 building block.

In the first proof-of-concept experiment, we could successfully produce 2 mM *n*-butanol, which corresponds to 148 mg L^−1^. Even with this prototypic approach, we could already achieve a 60-fold increase of the *n*-butanol yield compared with a recently reported cell-based route utilizing an engineered *Saccharomyces cerevisiae* (2.5 mg L^−1^) system (Steen et al., 2008). Nevertheless, recent studies present much higher *n*-butanol titer for cell-based approaches. The native *n*-butanol producer *C. acetobutylicum* achieved ~12 g L^−1^ (Harris et al., 2001; Lee et al., 2008), while different mutants reached 17.6 g L^−1^ (Jang et al., 2013) and 18.6 g L^−1^, respectively (Formanek et al., 1997). Engineered *E. coli* strains produced *n*-butanol with a broad product titer spectrum, ranging from ~500 mg L^−1^ (Atsumi et al., 2008; Nielsen et al., 2009) to 30 g L^−1^ (Shen et al., 2011). Compared with the cell-based achieved titer, our current *in vitro* system requires further improvements.

Using the first principles approach for optimization of our cell-free *n*-butanol cascade, we compared the kinetic properties of the required enzyme activities. With the exception of YqjM, kinetic properties for all other enzyme activities could be extracted from reference data. The exact kinetic properties of YqjM under aerobe conditions could not be determined due to side-reactions with molecular oxygen, which led to a non-productive reduction of the NADH pool (Sommer et al., 2013b). Therefore, experimental optimization procedures were based on ADH and PDC kinetic data.

The ADH *k*_cat_ for acetaldehyde (141.44 s^−1^) was 10-fold higher than for butyraldehyde (49.16 s^−1^) (Table 3) and the PDC *k*_cat_ for pyruvate (113 s^−1^) was in a similar order of magnitude to the corresponding ADH value for butyraldehyde (Chang et al., 2000). Considering the proline reaction rate at neutral pH and 50°C, we therefore restricted the enzyme activities initially to half of the proline reaction rate. The activities were therefore set to 0.25 U mL^−1^, while proline was set to 0.5 U mL^−1^ (Reaction 1). However, this approach led only to 0.8 mM *n*-butanol (Figure S1 in Supplementary Material). Finally, the reaction with the opposite combination of the enzyme activities (0.5 U mL^−1^) and proline reaction rate (0.25 U mL^−1^) led to the best result of 2 mM *n*-butanol.

The collective data in this study indicate two factors currently limiting *n*-butanol formation. A primary engineering target would be substrate preference of the ADH activity, as the kinetic properties showed a strong preference of acetaldehyde instead of butyraldehyde as a substrate, which results in undesired ethanol (4.9 mM) production, thereby reducing the *n*-butanol yield. However, acetaldehyde is an essential upstream intermediate of the *n*-butanol pathway and cannot be substituted. Therefore, a new or engineered ADH activity that favors higher aldehydes would be advantageous. As there are currently no ADH variants with increased butyraldehyde selectivity available, an extensive mutagenesis project possibly in combination with high content activity screening is required to generate the required enzyme activity.

Further pathway improvement could be achieved by replacing proline with a synthetic analog that provides even higher reaction rates at neutral pH and lower temperatures. However, this approach requires extended chemical synthesis and kinetic studies that are beyond the scope of study. Similarly, the issue of the long-term stability of the redox cofactor NADH still remains, which also could be solved by substituting NADH with a synthetic analogous of higher reactivity and stability (Kaufman, 1998; Ansell and Lowe, 1999; Oohora and Hayashi, 2014). The addition of alternative catalysts to enhance the reaction rate is therefore principally possible but has to be harmonized with the remaining enzyme activities of our designed reaction cascade.

Nonetheless, our cell-free *n*-butanol production route compares well with previously reported *in vitro n*-butanol technologies. For instance, the *n*-butanol production route reported by the Ohtake group applied almost the entire clostridial biosynthesis pathway. This pathway was reconstructed *in vitro* with a final *n*-butanol titer of 260 mg L^−1^ (Krutsakorn et al., 2013). To convert pyruvate to *n*-butanol, this approach required seven enzymes and two metabolic cofactors (NADH and CoA).

In contrast, our condensed *in vitro* concept requires only three enzymes, NADH as single cofactor and achieved 148 mg L^−1^ with a similar initial production rate of 0.01 μmol min^−1^ mL^−1^. Additionally, the combined biocatalytic *n*-butanol production pathway eliminates the need for any intermediate titration. Moreover, this pathway can be excellently integrated into our previously reported enzyme toolbox for *in vitro* solvent production (Guterl et al., 2012). A complete integration of the current reaction cascade would provide for direct conversion of glucose to *n*-butanol.

In summary, to complete our modular *in vitro* system to produce industrial solvents, we could successfully show the production of *n*-butanol *via* a new condensed pathway. Although the aldol condensation step restricted the overall reaction due to the limited reaction rate, we could compete with alternative cell-free *n*-butanol production concepts. The presented *in vitro n*-butanol production system demonstrates the capacity modern synthetic biotechnology methodologies and the synergies of chemoenzymatic technologies.

## Author Contributions

SR and TB conceived the study. SR, TB, DG, MH, FB, and VS planned the experimental work. SR, DG, MH, BS, FS, and JC selected, prepared, and analyzed enzyme reactions described in this study. All authors contributed to data interpretation, drafting, and refining the manuscript versions. SR, TB, DG, MH, and VS finalized the publishable manuscript. All authors agree to be accountable for the integrity of work presented in the current manuscript.

## Conflict of Interest Statement

The authors declare that the research was conducted in the absence of any commercial or financial relationships that could be construed as a potential conflict of interest.
