# Supplementary material for: In Vitro Bioconversion of Pyruvate to n-Butanol with Minimized Cofactor Utilization
Source: Front Bioeng Biotechnol. 2016 Oct 17;4:74. doi: 10.3389/fbioe.2016.00074 (PMC5066087; doi:10.3389/fbioe.2016.00074)
Supplement: Supplementary file 1 [file Image_1.PDF]

Supplementary Material

In vitro bioconversion of pyruvate to *n*-butanol with minimized cofactor utilization

Steven Reiße [1,2], Daniel Garbe [1], Bettina Sommer [1], Martina Haack [1], Fabian Steffler [3], Jörg Carsten[3], Frank Bohnen [2], Volker Sieber [3] and Thomas Brück [1,2]*

[1]Professorship of Industrial Biokatalysis, Department of Chemistry, Technical University of Munich, Garching, Germany

[2]B&B Sustainable Innovations GmbH, Köln, Germany

[3] Chair of Chemistry of Biogenic Resources, Straubing Center of Science, Technical University of Munich, Straubing, Germany

*** Correspondence:** Prof. Dr. Thomas Brück, Professorship of Industrial Biokatalysis, Department of Chemistry, Technical University of Munich, Lichtenbergstraße 4, 85748 Garching, Germany.

Email: brueck@tum.de

Supplementary Figure 1:

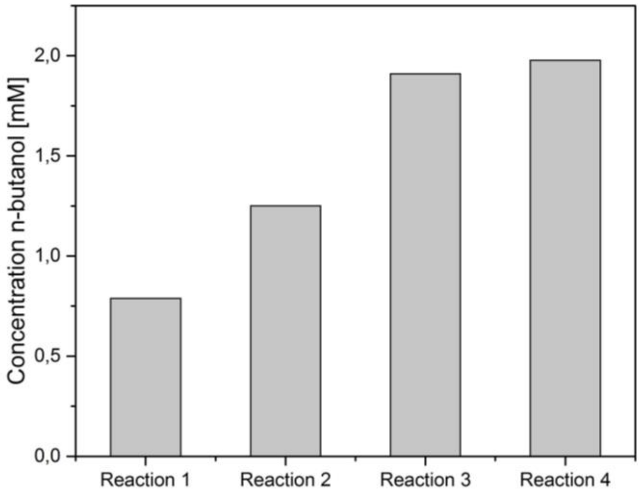

Figure S1. Test of the cell-free *n*-butanol production with various reaction designs. Reactions comprised 20 mM pyruvate, 0.05 mM FMN, 20 mM NADH, 2.5 mM $MgSO_4$ and 0.1 mM TPP in 50 mM HEPES buffer (pH 7) and were performed at 50 °C without stirring. The different reactions contained the following biocatalyst compositions: Reaction 1, 0.25 U ml^{-1} (PDC, YqjM, ADH) and 0.5 U ml^{-1} proline; Reaction 2, 0.25 U ml^{-1} (YqjM, ADH) and 0.5 U ml^{-1} (PDC, proline); Reaction 3, 0.5 U ml^{-1} (PDC, YqjM, ADH, proline); Reaction 4, 0.5 U ml^{-1} (PDC, YqjM, ADH) and 0.25 U ml^{-1} proline.
